# Stanniocalcin 2 Ameliorates Hepatosteatosis Through Activation of STAT3 Signaling

**DOI:** 10.3389/fphys.2018.00873

**Published:** 2018-07-09

**Authors:** Jiejie Zhao, Yang Jiao, Yuping Song, Jianmin Liu, Xiaoying Li, Huijie Zhang, Jialin Yang, Yan Lu

**Affiliations:** ^1^Department of Endocrinology and Metabolism, Shanghai Institute of Endocrine and Metabolic Diseases, Ruijin Hospital, Shanghai Jiao Tong University School of Medicine, Shanghai, China; ^2^Department of Endocrinology and Metabolism, Zhongshan Hospital, Fudan University, Shanghai, China; ^3^Department of Endocrinology and Metabolism, Minhang Branch, Zhongshan Hospital, Central Hospital of Minhang District, Shanghai Minhang Hospital, Fudan University, Shanghai, China; ^4^Department of Endocrinology and Metabolism, Nanfang Hospital, Southern Medical University, Guangzhou, China

**Keywords:** stanniocalcin 2, STAT3, non-alcoholic fatty liver disease, triglyceride metabolism, lipogenesis

## Abstract

Stanniocalcin 2 (STC2), a secreted glycoprotein hormone, regulates many biological processes, including cell proliferation, apoptosis, tumorigenesis, and atherosclerosis. However, its role in hepatic triglyceride metabolism remains unknown. In the present study, we found that expression levels of STC2 were significantly reduced in the livers of leptin-deficient and high fat diet-induced obese mice. Systemic administration of STC2 recombinant protein or adenovirus-mediated overexpression of STC2 markedly attenuated hepatosteatosis and hypertriglyceridemia in obese mice. At the molecular level, we found that STC2 activated the STAT3 signaling pathway to inhibit lipogenic gene expression. Consistently, *in vitro* studies further showed that inhibition of STAT3 signaling abolished the anti-steatotic effects of STC2. Together, our results revealed an important role of STC2 in the regulation of hepatic triglyceride metabolism, which might provide a potential therapeutic target for the treatment of fatty liver and related metabolic disorders.

## Introduction

Non-alcoholic fatty liver disease (NAFLD), defined as excess accumulation of triglycerides (TGs) in hepatocytes, has become the most common chronic liver condition and is estimated to impact at least 30% of Americans or Chinese (Browning et al., 2004; Williams et al., 2011; Wong et al., 2012). NAFLD can trigger a progressive cascade of liver disorders, ranging from hepatosteatosis to non-alcoholic steatohepatitis, liver cirrhosis, and even hepatocellular carcinoma (Farrell and Larter, 2006). Moreover, NAFLD is tightly associated with the development of type 2 diabetes, hypertension, atherosclerosis, and coronary heart disease (Marignani and Angeletti, 2002; Cohen et al., 2011).

Hepatosteatosis occurs when TG homeostasis is disrupted, due to increased TG synthesis and/or decreased TG clearance. In obesity-associated NAFLD, *de novo* lipogenesis (DNL) is increased, at least in part, by hyperinsulinemia as well as excess availability of carbohydrates (Lambert et al., 2014). Hepatic lipogenesis is mainly regulated by the transcription factor sterol regulatory element binding transcription protein 1c (*SREBP-1c*), which transcriptionally activates the expression of genes involved in DNL, including fatty acid synthetase (*FASN*), acetyl-CoA carboxylase (*ACC1*), and stearoyl-CoA desaturase-1 (*SCD1*). Indeed, increased hepatic expression levels of *SREBP-1c* and its target genes have been observed in obese rodents and humans (Shimomura et al., 1999; Horton et al., 2002).

Stanniocalcins (STC1 and STC2) were initially identified in bony fish as a calcium/phosphate-regulating hormone produced by the corpuscles of Stannius (Wagner et al., 1986). STC2 has the full-length of stanniocalcin sequence, while STC1 lacks a cysteine residue corresponding to Cys120 of STC2. Subsequent studies revealed that STC2 is ubiquitously expressed and acts as an endocrine, paracrine, or autocrine factor to regulate many biological processes, including tissue remodeling, cell survival, and stress responses (Law and Wong, 2010; Jepsen et al., 2016; Wu et al., 2017). Recently, increasing evidence highlighted its potential role in tumorigenesis, because its expression was markedly upregulated in several types of human malignancy, including stomach, colon, renal, and liver cancers (Meyer et al., 2009; Arigami et al., 2013; Chen et al., 2016; Wu et al., 2017). However, the role of STC2 in the regulation of hepatic TG homeostasis and in the pathogenesis of NAFLD remains unknown.

In the present study, we found that STC2 expression was reduced in the livers of leptin-deficient and high fat diet (HFD) -induced obese mice. Overexpression of STC2 significantly attenuated fatty liver and hypertriglyceridemia in obese mice through activation of the STAT3 signaling pathway. These findings revealed a vital role of STC2 in the regulation of hepatic TG homeostasis and suggested a promising therapeutic target for the related diseases.

## Materials and Methods

### Animal Experiments

Male C57BL/6 mice aged 8 weeks were purchased from the Shanghai Laboratory Animal Company (Shanghai, China). *ob/ob* mice were purchased from Nanjing Biomedical Research Institute of Nanjing University (Nanjing, Jiangsu Province, China). HFD-induced obese mice were maintained with free access to HFD (D12492; Research Diet, New Brunswick, NJ, United States) for 12 weeks, and control mice were fed with normal chow diet (NCD) (D12450B; Research Diet). STC2 recombinant protein was purchased from Shanghai Boyi Biotechnology Company (Shanghai, China). For systemic STC2 treatments, *ob/ob* mice received daily intraperitoneal (i.p.) injections of recombinant STC2 protein (0.5 mg/kg). Adenoviruses expressing murine *STC2* gene or green fluorescent protein (GFP) (Ad-STC2 and Ad-GFP) were constructed by Genechem Company (Shanghai, China). Overexpression of *STC2* or GFP in the liver of *ob/ob* mice was achieved by means of tail vein injection of Ad-STC2 or Ad-GFP [2 × 10^9^ plaque-forming units (pfu) for each mouse]. All animal experiments were conducted in accordance with the guidelines of Animal Care Committee of Shanghai Jiao Tong University School of Medicine.

### Cell Culture

Mouse primary hepatocytes (MPHs) were isolated from adult mice and maintained in hepatocyte medium (Sciencell, Carlsbad, CA, United States). HepG2 cells were cultured in DMEM (Gibco, Gaithersburg, MD, United States) containing 10% fetal bovine serum (FBS; Gibco), 100 IU/mL penicillin, and 100 μg/mL streptomycin. For the *in vitro* model of cellular steatosis, HepG2 cells were exposed to palmitic acid (PA) (200 μM) for 24 h to induce cellular TG accumulation, then were treated with recombinant STC2 protein (20 ng/mL) or vehicle control for another 24 h. To explore the potential signaling pathway affected by STC2, MPHs and HepG2 cells were starved in serum-free DMEM overnight and then treated with recombinant STC2 protein (20 ng/mL) for 1 h. To further investigate the role of STAT3 activation, HepG2 cells were pretreated with S31-201 (50 μM), a STAT3 inhibitor, to block STAT3 function, and then exposed to recombinant STC2 protein or vehicle control for 24 h. Cellular TG contents were extracted and determined using commercial kits (BioVision, Milpitas, CA, United States).

### Biochemical Measurements

Plasma or hepatic TGs and plasma total cholesterol (TC) were extracted and quantified using commercial kits (BioVision), according to the manufacturer’s instructions.

### Histological Analysis

For Hematoxylin and Eosin (H&E) staining, liver tissues were fixed overnight in 10% formalin, embedded in paraffin, and sectioned at 5 μm. Sections were subjected to standard H&E staining. For Oil Red O staining, liver tissues were fixed overnight in 4% paraformaldehyde, embedded in optimum cutting temperature compound, and cryosectioned. Frozen liver sections were stained with 0.15% Oil Red O according to standard procedures.

### RNA Isolation and qRT-PCR

Total RNA was isolated from cell lysates or liver tissues using TRIzol^^®^^ reagent according to the manufacturer’s instructions (Invitrogen, Shanghai, China). 2 μg of total RNA was reverse transcribed into cDNA using oligo-dT primers (Promega, Sunnyvale, CA, United States). Quantitative real-time PCR (qRT-PCR) was performed using SYBR^^®^^ Green Premix Ex Taq (Takara, Shiga, Japan) on a Light Cycler 480 (Roche, Basel, Switzerland) to quantify the gene transcripts of interest. The *36B4* gene was used as an internal reference for normalization. The primer sequences are listed in the **Table 1**.

**Table 1:** Description of primers used in real-time PCR.

Gene name	Species	Primer sequences (Forward 5′–3′)	Primer sequences (Reverse 5′–3′)
36B4	Mouse	AGATTCGGGATATGCTGTTGGC	TCGGGTCCTAGACCAGTGTTC
STC2	Mouse	CTGGGCCAGTTTGTGACCC	ACGTCATGCAAATCCCATGTAAA
SREBP-1c	Mouse	CTTTGGCCTCGCTTTTCGG	TGGGTCCAATTAGAGCCATCTC
FASN	Mouse	GGCTCTATGGATTACCCAAGC	CCAGTGTTCGTTCCTCGGA
ACC1	Mouse	AATGAACGTGCAATCCGATTTG	ACTCCACATTTGCGTAATTGTTG
SCD1	Mouse	TTCTTGCGATACACTCTGGTGC	CGGGATTGAATGTTCTTGTCGT
PPARα	Mouse	AACATCGAGTGTCGAATATGTGG	CCGAATAGTTCGCCGAAAGAA
CPT1α	Mouse	TGGCATCATCACTGGTGTGTT	GTCTAGGGTCCGATTGATCTTTG
ACOX1	Mouse	TAACTTCCTCACTCGAAGCCA	AGTTCCATGACCCATCTCTGTC
MCAD	Mouse	AACACAACACTCGAAAGCGG	TTCTGCTGTTCCGTCAACTCA
IL-6	Mouse	AGTTGCCTTCTTGGGACTGA	TCCACGATTTCCCAGAGAAC
TNF-α	Mouse	AGCCCCCAGTCTGTATCCTT	CTCCCTTTGCAGAACTCAGG
MCP1	Mouse	AGGTCCCTGTCATGCTTCTG	TCTGGACCCATTCCTTCTTG
Nrf2	Mouse	TCTTGGAGTAAGTCGAGAAGTGT	GTTGAAACTGAGCGAAAAAGGC
HO1	Mouse	AAGCCGAGAATGCTGAGTTCA	GCCGTGTAGATATGGTACAAGGA
α-SMA	Mouse	GTCCCAGACATCAGGGAGTAA	TCGGATACTTCAGCGTCAGGA
Col4a1	Mouse	CTGGCACAAAAGGGACGAG	ACGTGGCCGAGAATTTCACC
Col5a1	Mouse	CTTCGCCGCTACTCCTGTTC	CCCTGAGGGCAAATTGTGAAAA

### Western Blots

Liver tissues and HepG2 cells were lysed in radioimmunoprecipitation (RIPA) buffer containing protease and phosphatase inhibitors (Millipore, Billerica, MA, United States). 50 μg lysates were loaded onto 10% SDS-PAGE and transferred to polyvinylidenedifluoride (PVDF) membranes (Millipore, United States), which were blocked with 10% Bovine Serum Albumin and immunoblotted with antibodies at 4°C overnight. The antibodies used in western blots included, STAT3 (#12640; 1:1000, #9145; 1:1000, Cell Signaling Technology, Danvers, MA, United States), AKT (#13038; 1:1000, #4821; 1:1000, Cell Signaling Technology), JNK (#4668; 1:1000, #9258;1:1000, Cell Signaling Technology), Stanniocalcin 2 (#A302-369A; 1:500, Bethyl Laboratories, Montgomery, TX, United States), and GAPDH (KC-5G5; 1:5000, Aksomics, Shanghai, China). The proteins were visualized with Immobilon Western Chemiluminescent HRP Substrate (Millipore, United States) according to the manufacturer’s protocol.

### Insulin Tolerance Tests

*ob/ob* mice were injected i.p. with regular human insulin at a dose of 0.75 IU/kg body weight after fasting for 6 h. Blood glucose levels were monitored at the indicated time points using a portable blood glucose meter (LifeScan, Milpitas, CA, United States).

### Statistical Analysis

All values are shown as the mean ± standard error of the mean (SEM). Statistical differences were determined by two-tailed Student’s *t*-tests. Statistical significances were shown as ^∗^*P* < 0.05, ^∗∗^*P* < 0.01, or ^∗∗∗^*P* < 0.001.

## Results

### Hepatic STC2 Expression Was Reduced in Obese Mice

**FIGURE 1:**
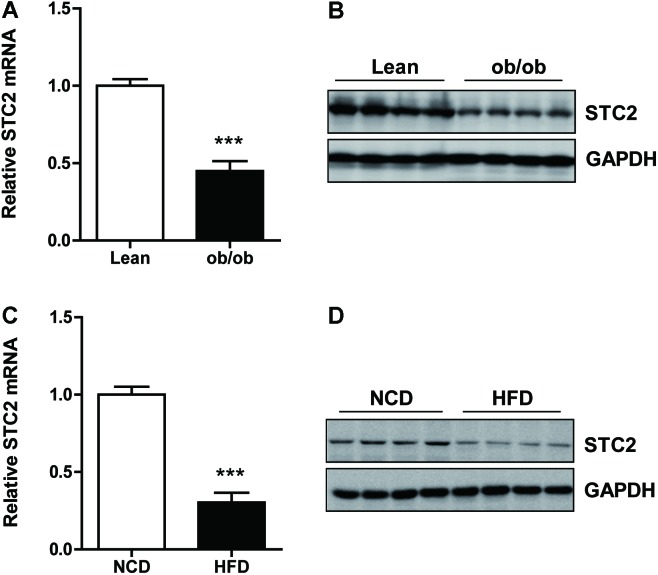
Hepatic STC2 expression was reduced in obese mice. **(A,B)** Hepatic Stanniocalcin 2 (STC2) mRNA and protein levels, determined by qRT-PCR and western blots, respectively, in *ob/ob* mice aged 8 weeks (*n* = 4–8). **(C,D)** Hepatic STC2 mRNA and protein levels, determined by qRT-PCR and western blots, respectively, in high fat diet (HFD) mice. The 8-week-old mice were fed normal chow diet (NCD) or a HFD for 12 weeks (*n* = 4–6). Data are expressed as the mean ± SEM. ^∗∗∗^*P* < 0.001.

To determine hepatic STC2 expression in obesity-associated NAFLD, we evaluated its mRNA and protein levels in the livers of *ob/ob* mice. Compared with lean mice, mRNA (**Figure 1A**) and protein (**Figure 1B**) levels of STC2 were significantly reduced in the livers of *ob/ob* mice. Similarly, the expression of hepatic STC2 was markedly decreased in mice fed a high fat diet (HFD) for 12 weeks as compared with mice fed a normal chow diet (NCD) (**Figures 1C,D**). These results indicated that downregulation of STC2 in the liver was a conserved feature of hepatosteatosis in obese mice.

### STC2 Alleviated Cellular TG Accumulation Through Suppression of *de Novo* Lipogenesis

**FIGURE 2:**
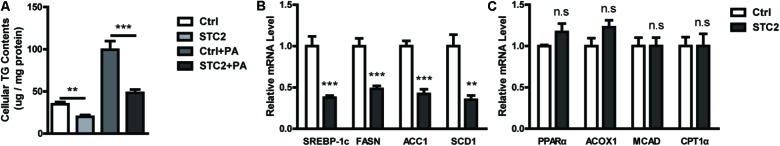
STC2 alleviated hepatic TG accumulation through suppression of *de novo* lipogenesis. **(A)** Cellular triglyceride (TG) contents in HepG2 cells. Cells were treated with recombinant STC2 protein (20 ng/mL) or phosphate-buffered saline (PBS) as a vehicle control for 24 h, with or without preincubated with palmitic acid (PA; 200 μM) for 24 h. **(B)** The mRNA levels of *SREBP-1c*, *FASN*, *ACC1*, and *SCD1* in HepG2 cells were measured. Cells were exposed to PA (200 μM) for 24 h, then were treated with recombinant STC2 protein (20 ng/mL) or vehicle control (PBS) for another 24 h. **(C)** The mRNA levels of *PPARα*, *ACOX1*, *MCAD*, and *CPT1α* in HepG2 were measured as in **(B)**. Data are expressed as the mean ± SEM. ^∗∗^*P* < 0.01, ^∗∗∗^*P* < 0.001.

To investigate the role of STC2 in hepatic TG metabolism, HepG2 cells were treated with recombinant STC2 protein or vehicle control. As a result, we found that cellular TG contents were decreased upon STC2 treatment (**Figure 2A**). In addition, STC2 dramatically inhibited the palmitate-induced cellular TG deposition (**Figure 2A**). To explore the molecular basis for the anti-steatotic effect of STC2, expression levels of genes involved in hepatic TG homeostasis were determined by quantitative real-time PCR. SREBP-1c, a master regulator of *de novo* lipogenesis, was significantly downregulated by STC2 treatment (**Figure 2B**). In parallel, expression levels of its downstream target genes, including fatty acid synthetase (*FASN*), acetyl-CoA carboxylase (*ACC1*), and stearoyl-CoA desaturase-1 (*SCD1*), were also reduced (**Figure 2B**). However, fatty acid oxidation-related genes, including peroxisome proliferator-activated receptor α (*PPARα*), carnitine palmitoyl transferase 1α (*CPT1α*), medium-chain acyl-CoA dehydrogenase (*MCAD*), and acyl-CoA oxidase (*ACOX1*), were not altered by STC2 treatment (**Figure 2C**).

### STC2 Activated the STAT3 Signaling Pathway to Alleviate Hepatosteatosis

**FIGURE 3:**
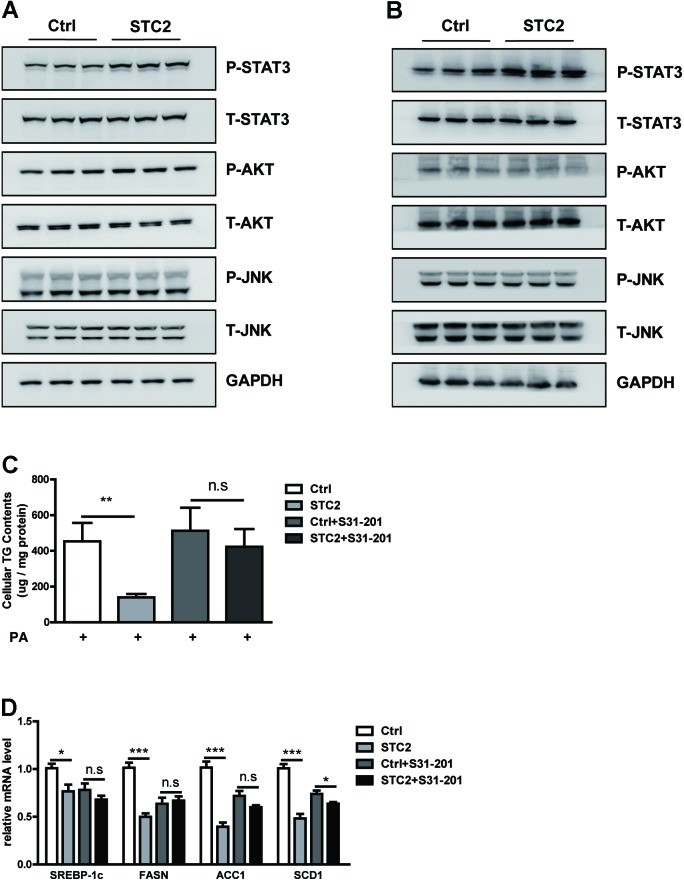
STC2 activated the STAT3 signaling pathway to alleviate hepatosteatosis. **(A,B)** Phosphorylated and total STAT3, AKT, and JNK in HepG2 cells **(A)** and MPHs **(B)** incubated with PBS or STC2 (20 ng/mL) protein for 1 h. Total STAT3, AKT, and JNK were used as loading controls. **(C)** Cellular TG contents in HepG2. Cells were treated with recombinant STC2 protein (20 ng/mL) or PBS vehicle control for 24 h, with or without preincubation with S31-201 (50 μM), a STAT3 inhibitor, for 2 h. **(D)** The mRNA levels of *SREBP-1c* and its target genes in HepG2 cells were measured as in **(C)**. Data are expressed as the mean ± SEM. ^∗^*P* < 0.05, ^∗∗^*P* < 0.01, ^∗∗∗^*P* < 0.001.

We analyzed the potential mechanism by which STC2 suppressed lipogenesis and alleviated TG accumulation in hepatocytes. We found that STC2 treatment resulted in marked activation of the STAT3 signaling pathway, as shown by enhanced phosphorylated STAT3, while the AKT and JNK pathways were unaffected (**Figures 3A,B**). To determine whether STAT3 activation was indispensable for the anti-steatotic effects of STC2, S31-201, a STAT3 inhibitor, was used to block STAT3 function. Our data showed that S31-201 abrogated the effects of STC2 on TG contents and lipogenic gene expression (**Figures 3C,D**).

### Systemic STC2 Treatment Ameliorated Hepatosteatosis in Obese Mice

**FIGURE 4:**
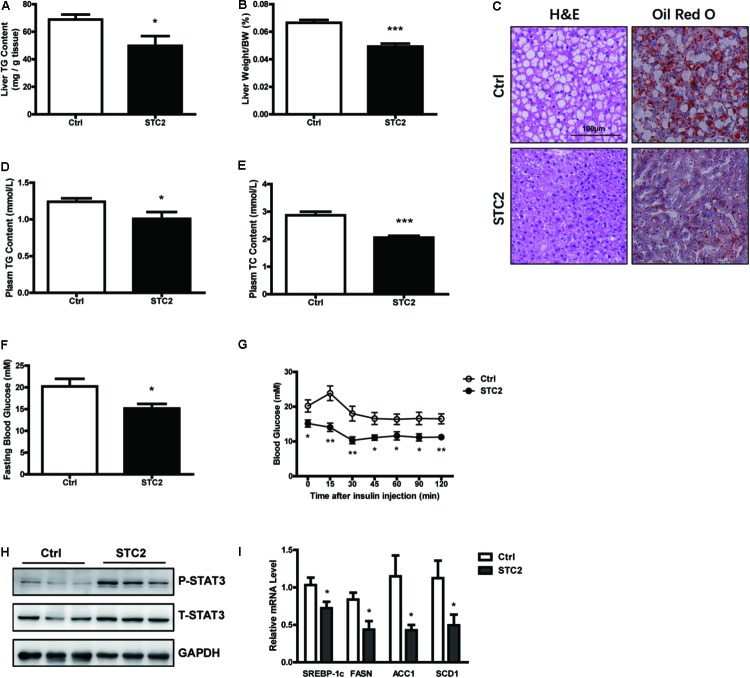
Systemic STC2 treatment ameliorated hepatosteatosis in obese mice. **(A,B)** Liver TG contents **(A)** and liver weight **(B)** in *ob/ob* mice intraperitoneally injected with recombinant STC2 protein (0.5 mg/kg) or vehicle control for 9 days (*n* = 7–8). **(C)** Representative histology (H&E, left) or Oil Red O (right) staining showing TG accumulation in livers from mice injected with STC2 protein versus vehicle control. Original magnification, 200×. **(D,E)** Plasm TG **(D)** and plasm TC **(E)** levels in mice. **(F,G)** Fasting blood glucose **(F)** and insulin tolerance test results **(G)** in mice. **(H)** Phosphorylated and total STAT3 in the livers of mice. Total STAT3 were used as loading controls. **(I)** The mRNA levels of hepatic *SREBP-1c* and its target genes in mice. Data are expressed as the mean ± SEM. ^∗^*P* < 0.05, ^∗∗^*P* < 0.01, ^∗∗∗^*P* < 0.001. H&E, Hematoxylin & Eosin; TG, triglyceride; TC, total cholesterol.

To elucidate the function of STC2 *in vivo*, *ob/ob* mice were injected i.p. with recombinant STC2 protein (0.5 mg/kg) once daily. A drastic decrease in hepatic TG contents (**Figure 4A**) and liver weight (**Figure 4B**) were observed in STC2-treated mice. Systemic STC2 administration also decreased the balloon cells and neutral lipid deposition in the livers of *ob/ob* mice as assessed by H&E and Oil Red O staining (**Figure 4C**). Plasma TG and total cholesterol (TC) levels were markedly reduced (**Figures 4D,E**). In parallel, STC2 partially ameliorated the inflammation, oxidative stress and fibrosis characteristics of *ob/ob* mice (**Supplementary Figures S1A–C**). Plasma ALT and AST levels were also significantly decreased in STC2-treated mice (**Supplementary Figures S1D,E**). In addition, fasting blood glucose levels and insulin sensitivity were dramatically improved upon STC2 treatment (**Figures 4F,G**). Consistently, STC2 also activated the STAT3 pathway and reduced the expression of SREBP-1c and its target genes in the liver (**Figures 4H,I**).

### Hepatic STC2 Overexpression Attenuated Fatty Livers in *ob/ob* Mice

**FIGURE 5:**
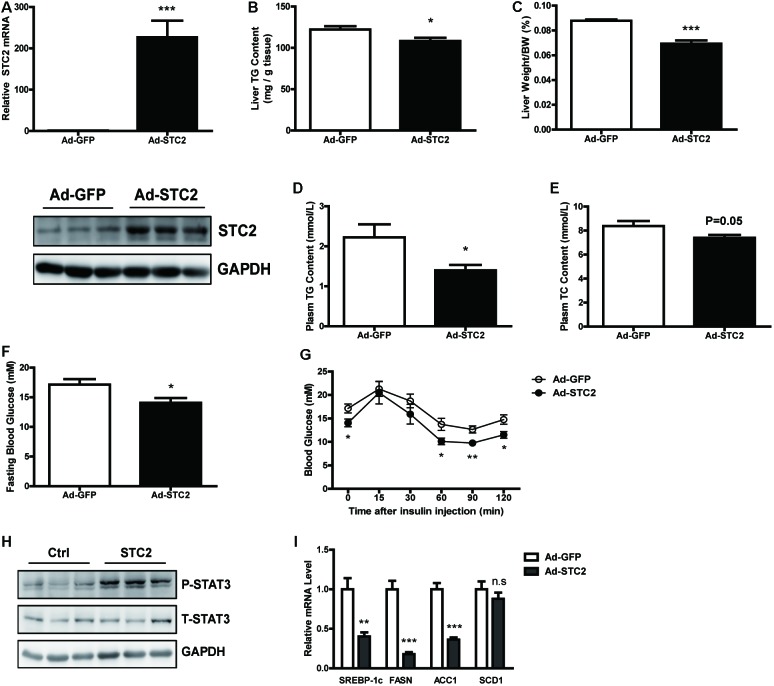
Hepatic STC2 overexpression attenuated fatty liver in *ob/ob* mice. **(A)** The mRNA and protein levels of STC2 in *ob/ob* mice injected with adenovirus containing GFP or the STC2 gene (*n* = 7–8). **(B,C)** Liver TG contents **(B)** and liver weights **(C)** in mice. **(D,E)** Plasm TG **(D)** and plasm TC **(E)** levels in mice. **(F,G)** Fasting blood glucose **(F)** and insulin tolerance test results **(G)** in mice. **(H)** Phosphorylated and total STAT3 in the livers of mice. Total STAT3 were used as loading controls. **(I)** The mRNA expressions of hepatic *SREBP-1c* and its target genes in mice. Data are expressed as the mean ± SEM. ^∗^*P* < 0.05, ^∗∗^*P* < 0.01, ^∗∗∗^*P* < 0.001. TG, triglyceride; TC, total cholesterol.

Next, *STC2* gene was overexpressed in the livers of *ob/ob* mice by delivering an adenovirus via tail vein injection. Increased mRNA and protein levels of STC2 in the liver were confirmed by qRT-PCR and western blots, respectively, while its expression in white adipose tissues were not affected (**Figure 5A** and **Supplementary Figures S2A,B**). Hepatic overexpression of STC2 significantly decreased liver TG content (**Figure 5B**) and liver weight (**Figure 5C**). Plasma TG levels were markedly reduced (**Figure 5D**), and a declining tendency in plasma TC levels was also observed (**Figure 5E**). Consistently, hepatic overexpression of STC2 partially ameliorated the inflammation, oxidative stress and fibrosis characteristics of *ob/ob* mice (**Supplementary Figures S2C–E**). Plasma ALT levels were significantly reduced, while AST levels were not affected in STC2-overexpressed *ob/ob* mice (**Supplementary Figures S2F,G**). In addition, fasting hyperglycemia and insulin resistance were improved by STC2 overexpression in *ob/ob* mice (**Figures 5F,G**). At the molecular level, overexpression of STC2 also induced hepatic STAT3 activation and reduced the expression of *de novo* lipogenesis related genes (**Figures 5H,I**).

## Discussion

Previous studies have shown that STC2 regulates many biological processes, including tissue remodeling, cell survival, stress responses, and tumorigenesis. However, little is known about its function in metabolic homeostasis. Interestingly, STC2 knockout mice were reported to present with deregulated glycemia when they were fed with a hypercaloric diet (López et al., 2017). Enhanced glucagon immunostaining in the islet cells and elevated circulating glucagon levels were observed in STC2-null mice (López et al., 2017).

In the present study, we identified a novel role of STC2 in the regulation of hepatic TG homeostasis. Systemic STC2 administration or liver-specific overexpression of STC2 significantly ameliorated TG accumulation in obese mice. At the molecular level, it has been shown that STC2 can modulate multiple signaling pathways, such as PI3K/AKT, ERK1/2, in tumor cells and osteoblast (Zhou et al., 2016; Yang et al., 2017). However, our data showed that the AKT and JNK pathways were unaffected in STC2-treated hepatocytes. Therefore, the role and downstream signaling pathways of STC2 might be tissue or cell-specific, which needs further investigations in future studies.

In addition, studies using genetically engineered mice have shown the significance of the STAT3 signaling pathway in the pathophysiology of fatty liver. The anti-steatotic effects of STAT3 were mediated, at least in part, via transcriptional suppression of SREBP-1c and subsequent repression of hepatic *de novo* lipogenesis (Ueki et al., 2004). As a result, disruption of STAT3 or its upstream molecule gp130 in hepatocytes exacerbated fatty liver induced by a high fat diet (Inoue et al., 2004), alcohol-containing diet (Horiguchi et al., 2008), or a choline deficient ethionine-supplemented diet (Kroy et al., 2010). Overexpression of constitutively activated *STAT3* attenuated high fat diet-induced fatty livers (Inoue et al., 2004). Consistently, genetic variants in *STAT3* were associated with non-alcoholic fatty liver disease in humans (Sookoian et al., 2008). Furthermore, several cytokines are known to activate the STAT3 pathway to regulate hepatic TG homeostasis. For instance, treatment with IL-6 ameliorated fatty liver by inducing STAT3 phosphorylation in *ob/ob* and HFD- induced obese mice (Hong et al., 2004), while deletion of IL-6 or hepatic STAT3 resulted in steatosis and hepatocellular damage in IL-10 knockout mice (Miller et al., 2011). In addition, IL-22 ameliorated non-alcoholic and alcoholic fatty livers through activation of the STAT3 pathway in hepatocytes (Ki et al., 2010; Yang et al., 2010). Here, in our studies we found that plasma IL-22 contents were reduced by STC2 treatment or overexpression (**Supplementary Figures S3A,B**). Previous studies have shown that inflammatory signaling, including NF-κB and AP-1/JunD, are involved in IL-22 production (Ouyang et al., 2011; Ahlfors et al., 2014; Fumagalli et al., 2016). Since STC2 treatment can reduce hepatic inflammation in obese mice, we speculate that reduction of IL-22 in STC-2 treated mice might be attributed to decreased inflammation.

## Conclusion

Our findings demonstrated that STC2 was an important metabolic regulator in the liver. STC2 ameliorated hepatosteatosis and hypertriglyceridemia in obese mice, mainly through activation of the STAT3 signaling pathway. Thus, STC2 may be a promising therapeutic target for fatty liver and dyslipidemia.

## Author Contributions

YL, JY, HZ, and XL conceived the project. YL, JZ, and YJ designed the experiments. JZ, YJ, and YS performed the experiments and statistical analysis. JZ, YJ, and YL drafted the manuscript. YJ, YL, JL, and XL handled funding and supervision. All authors reviewed the manuscript.

## Conflict of Interest Statement

The authors declare that the research was conducted in the absence of any commercial or financial relationships that could be construed as a potential conflict of interest. The reviewer DV and handling Editor declared their shared affiliation.

## Supplementary Material

FIGURE S1 Systemic STC2 treatment ameliorated hepatosteatosis in obese mice. **(A)** Relative mRNA levels of IL-6, TNF-α, MCP1 in the livers of two groups. **(B)** Relative mRNA levels of Nrf2, HO1 in the livers of two groups. **(C)** Relative mRNA levels of hepatic α-SMA, Col4a1, Col5a1in two groups. **(D,E)** Plasma ALT and AST levels in two groups. ^∗^*P* < 0.05, ^∗∗^*P* < 0.01, ^∗∗∗^*P* < 0.001. MCP1, Chemokine (C-C motif) ligand 2 (CCL2); Nrf2, nuclear factor, erythroid derived 2, like 2; HO1, heme oxygenase 1; Col4a1, collagen, type IV, alpha 1; Col5a1, collagen, type V, alpha 1.

FIGURE S2 Hepatic STC2 overexpression attenuated fatty liver in ob/ob mice. **(A,B)** Relative mRNA and protein expression of STC2 in white adipose tissues. **(C)** Relative mRNA levels of IL-6, TNF-α, MCP1 in the livers of two groups. **(D)** Relative mRNA levels of Nrf2, HO1 in the livers of two groups. **(E)** Relative mRNA levels of hepatic α-SMA, Col4a1, Col5a1 in two groups. **(F,G)** Plasma ALT and AST levels in two groups. ^∗^*P* < 0.05, ^∗∗^*P* < 0.01, ^∗∗∗^*P* < 0.001.

FIGURE S3 Plasma IL-22 contents in systemic STC2-treated and hepatic STC2 overexpressed ob/ob mice. **(A,B)** Plasma IL-22 contents in STC2-treated ob/ob mice **(A)** or hepatic STC2 overexpressed ob/ob mice **(B)** by ELISA kit. ^∗^*P* < 0.05, ^∗∗^*P* < 0.01, ^∗∗∗^*P* < 0.001.
