# Supplementary figures and images for: Stanniocalcin 2 Ameliorates Hepatosteatosis Through Activation of STAT3 Signaling
Source: Front Physiol. 2018 Jul 9;9:873. doi: 10.3389/fphys.2018.00873 (PMC6046442; doi:10.3389/fphys.2018.00873)

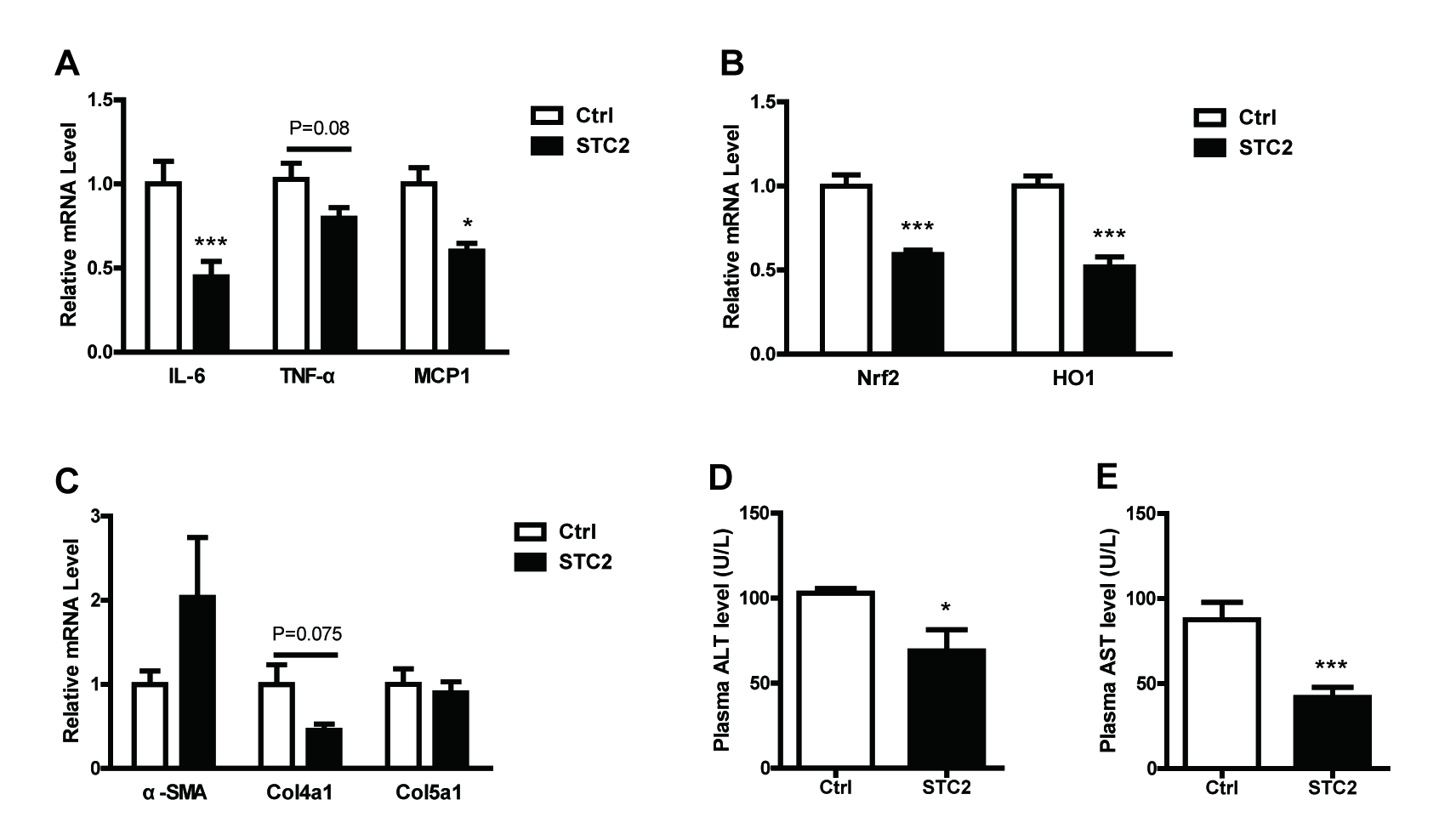

Supplement: FIGURE S1 — Systemic STC2 treatment ameliorated hepatosteatosis in obese mice. (A) Relative mRNA levels of IL-6, TNF-α, MCP1 in the livers of two groups. (B) Relative mRNA levels of Nrf2, HO1 in the livers of two groups. (C) Relative mRNA levels of hepatic α-SMA, Col4a1, Col5a1in two groups. (D,E) Plasma ALT and AST levels in two groups. ∗P < 0.05, ∗∗P < 0.01, ∗∗∗P < 0.001. MCP1, Chemokine (C-C motif) ligand 2 (CCL2); Nrf2, nuclear factor, erythroid derived 2, like 2; HO1, heme oxygenase 1; Col4a1, collagen, type IV, alpha 1; Col5a1, collagen, type V, alpha 1. [file Image_1.TIF]

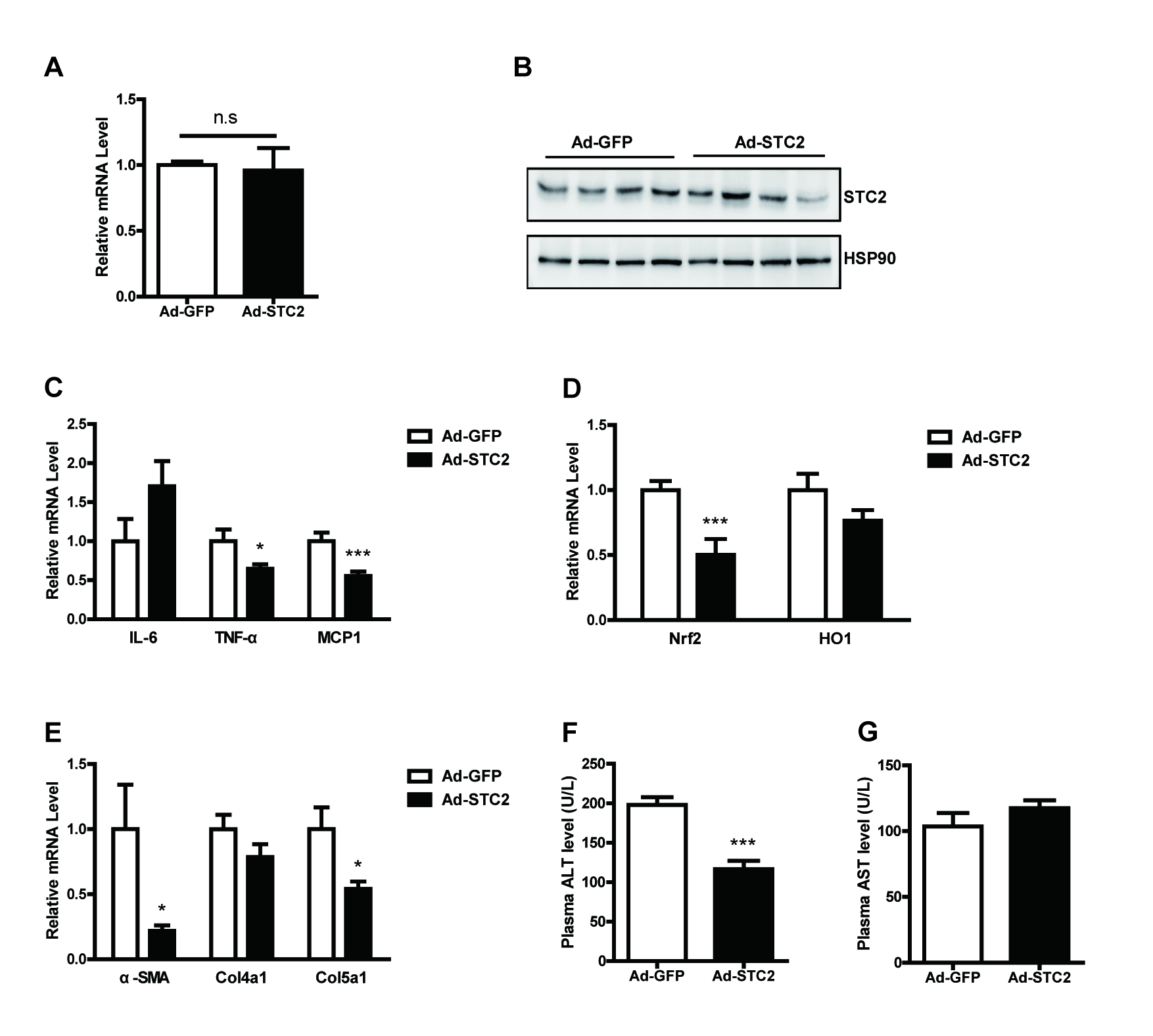

Supplement: FIGURE S2 — Hepatic STC2 overexpression attenuated fatty liver in ob/ob mice. (A,B) Relative mRNA and protein expression of STC2 in white adipose tissues. (C) Relative mRNA levels of IL-6, TNF-α, MCP1 in the livers of two groups. (D) Relative mRNA levels of Nrf2, HO1 in the livers of two groups. (E) Relative mRNA levels of hepatic α-SMA, Col4a1, Col5a1 in two groups. (F,G) Plasma ALT and AST levels in two groups. ∗P < 0.05, ∗∗P < 0.01, ∗∗∗P < 0.001. [file Image_2.TIF]

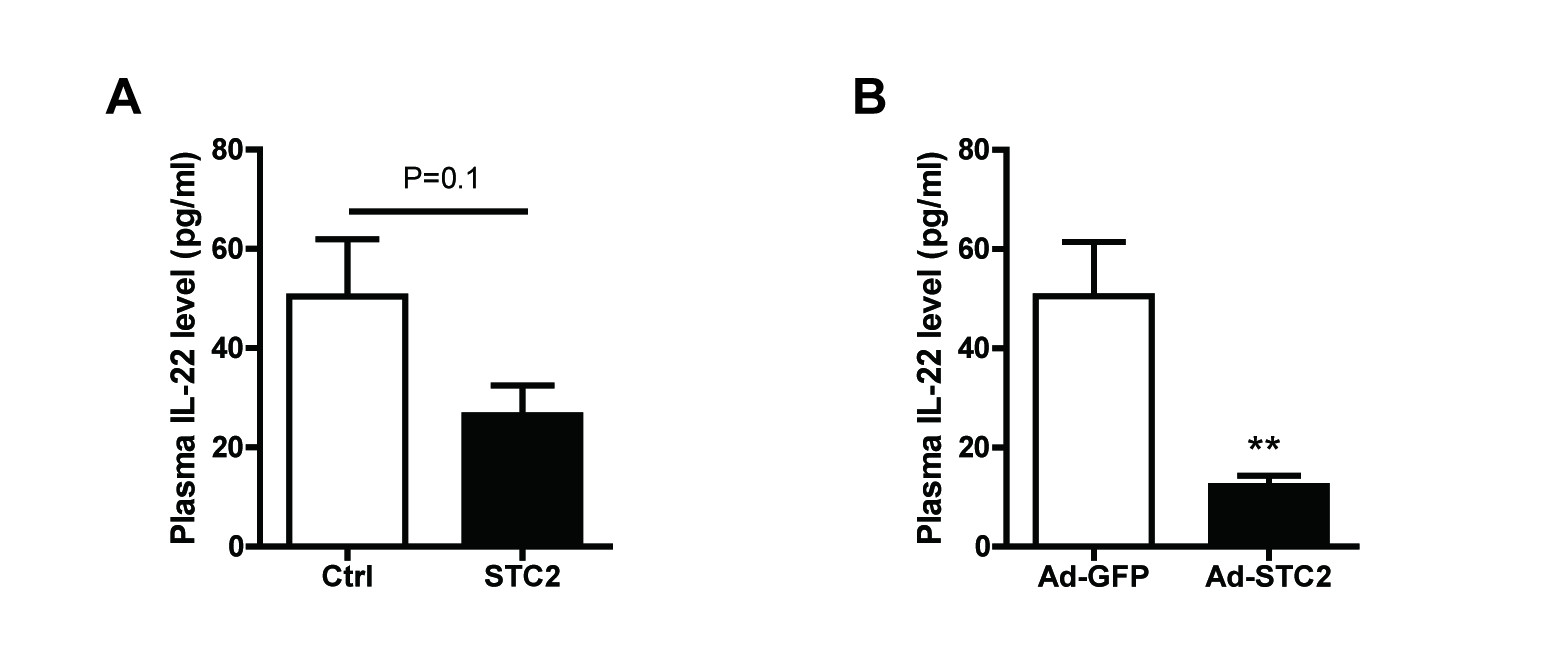

Supplement: FIGURE S3 — Plasma IL-22 contents in systemic STC2-treated and hepatic STC2 overexpressed ob/ob mice. (A,B) Plasma IL-22 contents in STC2-treated ob/ob mice (A) or hepatic STC2 overexpressed ob/ob mice (B) by ELISA kit. ∗P < 0.05, ∗∗P < 0.01, ∗∗∗P < 0.001. [file Image_3.TIF]
